# Nematicidal Volatiles from *Bacillus atrophaeus* GBSC56 Promote Growth and Stimulate Induced Systemic Resistance in Tomato against *Meloidogyne incognita*

**DOI:** 10.3390/ijms22095049

**Published:** 2021-05-10

**Authors:** Muhammad Ayaz, Qurban Ali, Ayaz Farzand, Abdur Rashid Khan, Hongli Ling, Xuewen Gao

**Affiliations:** 1 Key Laboratory of Integrated Management of Crop Diseases and Pests, Ministry of Education, Department of Plant Pathology, College of Plant Protection, Nanjing Agricultural University, Nanjing 210095, China; m.ayazbiotech@gmail.com (M.A.); qurbanalirattar@webmail.hzau.edu.cn (Q.A.); malix.477@hotmail.com (A.R.K.); 2 Department of Plant Pathology, University of Agriculture, Faisalabad P.O. Box 38040, Pakistan; ayaz.farzand@uaf.edu.pk; 3 Shandong Vland Biotechnology Co., Ltd., Binzhou 251700, China; linghl@vlandgroup.com

**Keywords:** GC-MS analysis, biocontrol, methyl isovalerate, 2-undecanone, mortality, nematicidal volatiles, oxidative stress

## Abstract

*Bacillus* volatiles to control plant nematodes is a topic of great interest among researchers due to its safe and environmentally friendly nature. *Bacillus* strain GBSC56 isolated from the Tibet region of China showed high nematicidal activity against *M. incognita*, with 90% mortality as compared with control in a partition plate experiment. Pure volatiles produced by GBSC56 were identified through gas chromatography and mass spectrometry (GC-MS). Among 10 volatile organic compounds (VOCs), 3 volatiles, i.e., dimethyl disulfide (DMDS), methyl isovalerate (MIV), and 2-undecanone (2-UD) showed strong nematicidal activity with a mortality rate of 87%, 83%, and 80%, respectively, against *M. incognita*. The VOCs induced severe oxidative stress in nematodes, which caused rapid death. Moreover, in the presence of volatiles, the activity of antioxidant enzymes, i.e., SOD, CAT, POD, and APX, was observed to be enhanced in *M. incognita*-infested roots, which might reduce the adverse effect of oxidative stress-induced after infection. Moreover, genes responsible for plant growth promotion *SlCKX1*, *SlIAA1*, and *Exp18* showed an upsurge in expression, while *AC01* was downregulated in infested plants. Furthermore, the defense-related genes (*PR1*, *PR5*, and *SlLOX1*) in infested tomato plants were upregulated after treatment with MIV and 2-UD. These findings suggest that GBSC56 possesses excellent biocontrol potential against *M. incognita*. Furthermore, the study provides new insight into the mechanism by which GBSC56 nematicidal volatiles regulate antioxidant enzymes, the key genes involved in plant growth promotion, and the defense mechanism *M. incognita*-infested tomato plants use to efficiently manage root-knot disease.

## 1. Introduction

Plant-parasitic nematodes (PPNs) severely affect the growth and productivity of several crops [1]. To date, over 4100 species of PPNs have been documented in the literature [2]. Amongst these PPNs, the root-knot nematodes (*Meloidogyne* spp.) are the most notorious pathogens, causing about half of the total losses incurred to crops, estimated to cost over USD 75 billion annually [1,3,4]. Among *Meloidogyne* spp., *Meloidogyne incognita* have a broad-host range, high reproduction potential, and capability to cause complex diseases in synergy with other soil-borne pathogens, e.g., fungi [5], making it one of the most dangerous plant-parasitic nematodes [6]. The vascular tissue of the host roots is greatly damaged by the nematodes, resulting in root galling, imbalanced water utilization, reduced nutrient uptake, and affecting the photosynthetic process, which leads to symptoms such as wilting, yield reduction, and stunted growth [7]. Although chemical nematicidals are effective in controlling *M. incognita,* their adverse effects on the environment and human health prevent their extensive use [8]. Currently, researchers are working to find new directions and alternates for safe and environment-friendly strategies to overcome the losses caused by plant pathogens [9,10]. The use of biological control microorganisms or their volatile organic compounds (VOCs) provides strong nematicidal activity to alleviate the risk of root-knot disease [11]. Bacterial VOCs are less toxic to human health and more cost-effective as well. Therefore, the identification of different VOCs attracts significant attention [12].

Plant growth-promoting rhizobacteria (PGPRs) have been described as being able to fortify plant development by regulating plant hormones, such as auxins, gibberellins, cytokinin, and ethylene [13]. However, research on plant hormones’ interaction with *Bacillus* VOCs that results in efficient growth is at the initial stages [14]. The first reported two VOCs (2,3- butanediol and acetoin) produced by *Bacillus megaterium* XTBG34 promoted the growth of *Arabidopsis thaliana* plants by 2-fold after 15 days of treatment [15]. More recently 13-tetradecadien1-ol, 2-butanone, and 2-methyl-n-1-tridecene, produced by *Pseudomonas fluorescens* SS101, enhanced the growth of *Nicotiana tabacum* [16]. The previous finding over the past decade has described that *Bacillus* volatile can interact with phytohormones, resulting in stimulation of plant growth [14,17]. The key role of phytohormones and the genes involved in different signaling pathways needs to be studied in detail to explore the mechanism of plant growth promotion in the presence of *Bacillus* volatiles [18,19]. Few volatiles are reported to alter the relative expression levels of most important genes relating to expansin (*Exp2*, *Exp9*, *Exp18*), auxin (*SlIAA1*, *SlIAA3*), ethylene biosynthesis (*ACO1*), and cytokinin *(SlCKX1)*, which are considered as marker genes in the growth promotion mechanism of the plant [14,20,21]. Hence, the major role of volatiles in regulating genes has attracted great attention of researchers who seek to explore nematicidal VOCs that have the potential to promote plant growth and stimulate induced systemic resistance; moreover, the topic has become of great interest in the field of plant pathology for the purpose of better managing the diseases caused by different nematodes [22,23].

Plant growth-promoting rhizobacteria (PGPR) produce VOCs that induce systemic resistance against different pathogens [1,15,16]. PGPRs produce VOCs under normal conditions and are highly active against pathogens, even at low concentrations [24,25]. These VOCs play a beneficial role in three ways: they control plant pathogens, they stimulate plant growth, and they induce systemic resistance [13,26]. The plant defense system possesses various pathways to induce an immune response against pathogens through coordinated signaling networks that include reactive oxygen species (ROS), phytohormones, and defense-linked genes [27]. Volatiles play a crucial role in triggering the immune response through jasmonic acid (JA), abscisic acid (i.e., ABA and brassinosteroids (BRs), ethylene (ET), salicylic acid (SA), etc.). Recent studies have shown that JA-induced defense enhances resistance against root-knot nematodes (RKN) and regulates antioxidant enzymes and photosynthetic processes [28]. Moreover, SA used as a soil drenching treatment boosts induced systemic resistance (ISR) that limits the invasion of the second stage J2 juveniles of *M. incognita* in tomato roots [29]. In rice (*Oryza sativa*) plants, the JA pathway plays a vital role in inducing systemic resistance against RKN [30]. These phytohormones are essential components of the plant defense against nematode infection [31,32]. The defense-related genes, i.e., *PR1* involved in SA signaling, lipoxygenase gene *SlLOX1*, and *PR5*, mostly induced in nematode infection are considered as marker genes in the defense mechanism of tomato plants under biotic stress. However, the regulation of these genes by nematicidal VOCs are rarely studied and their interaction with VOCs to induce a strong defense response in plants against nematodes needs to be explored [10,33].

PGPRs in direct contact are extensively studied for their potential to promote plant growth and to stimulate ISR against nematodes in infested plants [34]. The ISR emerged as an important mechanism by which selected plant growth-promoting bacteria and fungi in the rhizosphere prime the whole plant body for enhanced defense against a broad range of pathogens [35]**. **However, microorganisms that produce volatile substances in soil or other growth materials received little attention for nematicidal activity. In our lab, we have extensively studied *Bacillus* strains obtained from Tibet, China, for their antagonistic activity against pathogenic fungi and bacteria; still, little is known about their nematicidal activity against *M. incognita* that causes root-knot disease, which is becoming a serious threat to important crops globally. The main objective of the current research was to identify efficient nematicidal volatiles produced by *Bacillus* sp. strain GBSC56 against *M. incognita,* an important nematode that causes root-knot disease in various crops. Moreover, the study provides new insight into the mechanism of nematicidal volatiles by regulating the antioxidant enzymes and the key genes involved in plant growth promotion and the defense mechanism that *M. incognita*-infested plants use to efficiently manage root-knot disease.

## 2. Results

### 2.1. Nematicidal Activity of GBSC56-VOCs and ROS Induction in Meloidogyne incognita

**Figure 1:**
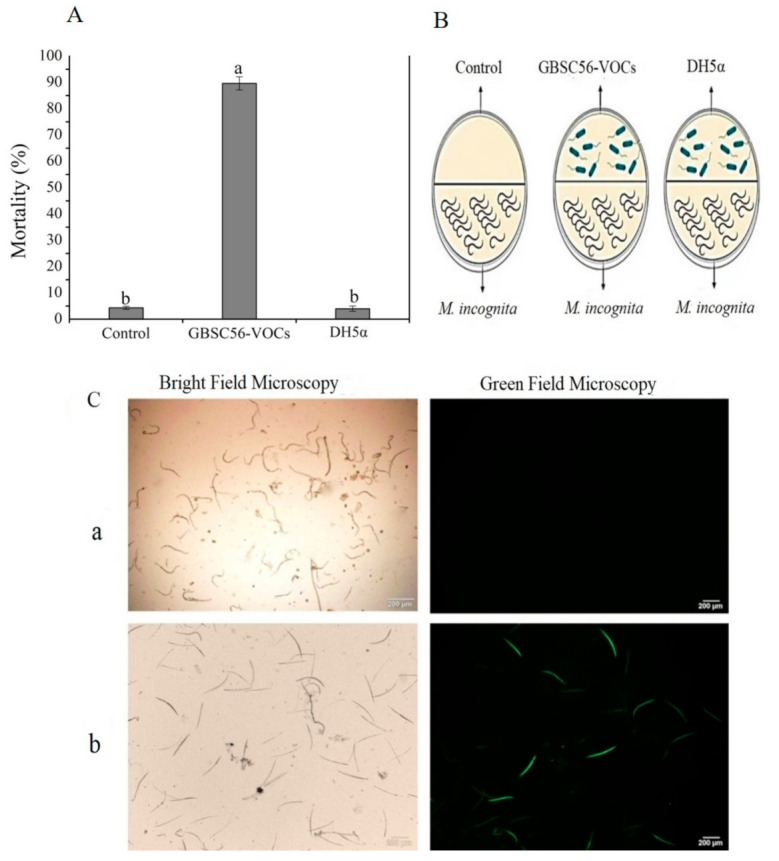
Nematicidal activity of GBSC56-VOCs compared with control and DH5α. (**A**) Graphical detail of nematicidal activity of GBSC56-VOCs. (**B**) Partition plate assay to find the nematicidal activity of GBSC56. (**C**) ROS induction in nematodes pre-exposed to GBSC56-VOCs (**b**) caused severe oxidative damage as compared with control (**a**). Error bar represents the mean standard deviation of each treatment repeated three times with three replicates. The small letters above the columns indicate significant difference. The significant difference among the treatments was calculated through the Tukey HSD test at *p* ≤ 0.05.

GBSC56 was evaluated for its nematicidal activity against *M. incoginta* in vitro through a partition plate experiment. After 48 h of incubation at 25 °C, GBSC56 showed strong nematicidal activity with a mortality rate of 90% as compared with control (LB or DH5α). The GBSC56-VOCs gradually reduced the movement of nematodes after 24 h and the nematodes mostly stopped moving completely after 48 h. The nematodes were considered dead if they were unable to regain movement after being touched with a needle under the microscope. The dead nematodes were then studied for oxidative stress. GBSC56-VOCs killed nematodes by inducing ROS, which is an indication of severe damage, as shown in Figure 1.

### 2.2. In Vitro Plant Growth Promotion by GBSC56-VOCs

**Figure 2:**
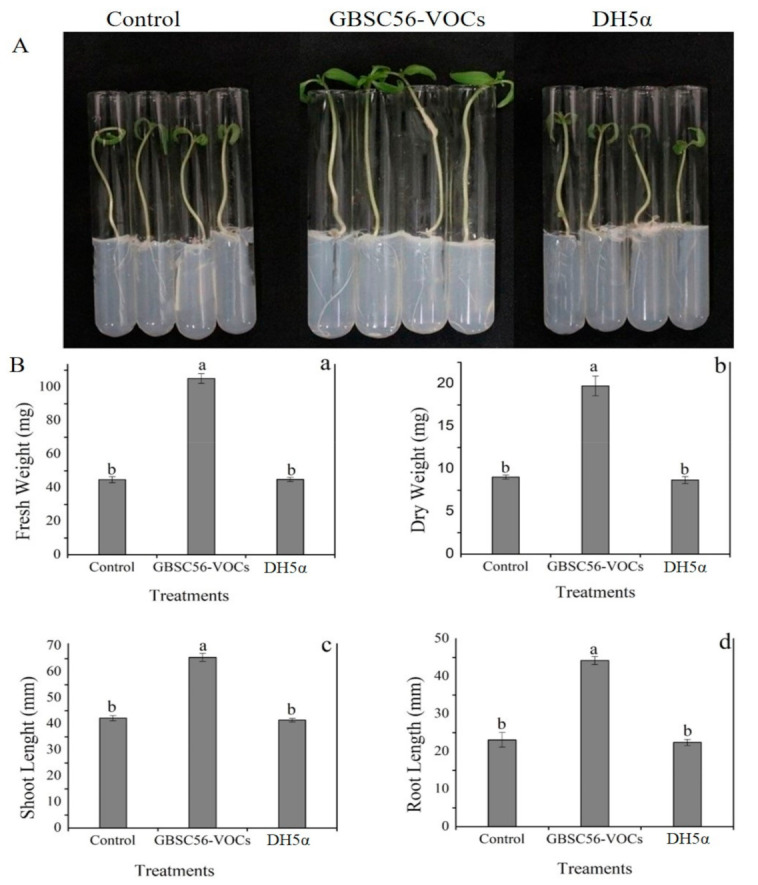
Seeds of tomato after surface sterilization were placed per each test tube and the resulting seedlings were grown in the presence of 20 µl of GBSC56 culture (10^7^ CFU/mL) in an airtight jar incubated at 25 °C for 12 days. The effect of GBSC56-VOCs on seedlings was observed visually (**A**) and calculated by the difference (**B**) in (**a**) fresh weight, (**b**) dry weight, (**c**) shoot length, and (**d**) root length after exposure to GBSC56-VOCs. Error bar represents the mean standard deviation of each treatment. The small letters above the columns indicate significant difference. The significant difference among the treatments was calculated through the Tukey HSD test at *p* ≤ 0.05.

Seeds of tomato after surface sterilization were placed per each test tube, and the resulting seedlings were grown in the presence of 20 µL of GBSC56 culture (colony-forming unit 10^7^ CFU/mL) in an airtight jar incubated at 25 °C for 12 days. A significant increase was visually seen in shoot length (32%), root length (29%), fresh weight (38%), and dry weight (24%) of tomato seedlings exposed to GBSC56-VOCs as compared with *Escherichia coli* (*E. coli)* (DH5α), as shown in Figure 2.

### 2.3. Identification of Pure VOCs Produced by GBSC56 Through SMPE-GC-MS Analysis

**Figure 3:**
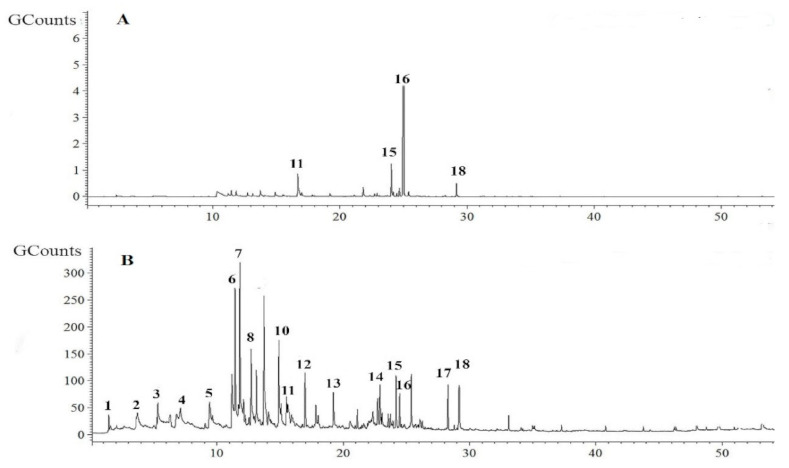
The GC chromatogram of the possible GBSC56 volatiles was identified through GC-MS analysis. Similar VOCs in the control are excluded. (**A**) The chromatograph of VOCs produced in Control. (**B**) The VOCs produced by GBSC56. The VOCs were selected in a time duration of 0–30 min. The numbering represents peaks for different VOCs. The pure possible VOCs identified are (1) cyclopentene (CP), (2) dimethyl disulfide (DMDS), (3) methyl isovalerate (MIV), (4) butanoic acid, 3-methyl, ethyl ester (BMEE), (5) ethyl tiglate (ET), (6) ethyl-2-methyl butyrate (EMB), (7) 2- methylheptonic acid (MHA), (8) octanoic acid 2-methyl (OAM), (14) 2-undecanone (2-UD), (17) pentadecane (PD).

GBSC56 with the potential to promote plant growth and strong nematicidal activity against *M. incognita* enabled us to hypothesize that the strain might produce VOCs that promote plant growth with the potential to kill *M. incognita.* Therefore, 20 μL culture of GBSC56 was grown on LB medium in a 100 μL vial at 28 °C for 4 days for the purpose of obtaining VOCs for SMPE-GC-MS analysis. Mass spectra data of the possible VOCs produced by the strain were compared with the data in the NIST/EPA/NIH Mass Spectrum Library. The possible VOCs with relatively high peak areas, e.g., ≥1%, that were not similar to the control were identified from the GBSC56 strain. The VOCs were selected for a time duration of 0–30 min. The numbering represents peaks for different VOCs. The possible VOCs identified were, i.e., cyclopentene (CP), dimethyl disulfide (DMDS), methyl isovalerate (MIV), butanoic acid, 3-methyl, ethyl ester (BMEE), ethyl tiglate (ET), ethyl-2-methyl butyrate (EMB), 2-methylheptonic acid (MHA), octanoic acid 2-methyl (OAM), 2-undecanone (2-UD), and pentadecane (PD), as shown in Figure 3.

### 2.4. Evaluation of Each Pure VOCs for Their Nematicidal Activity against Meloidogyne incognita

**Figure 4:**
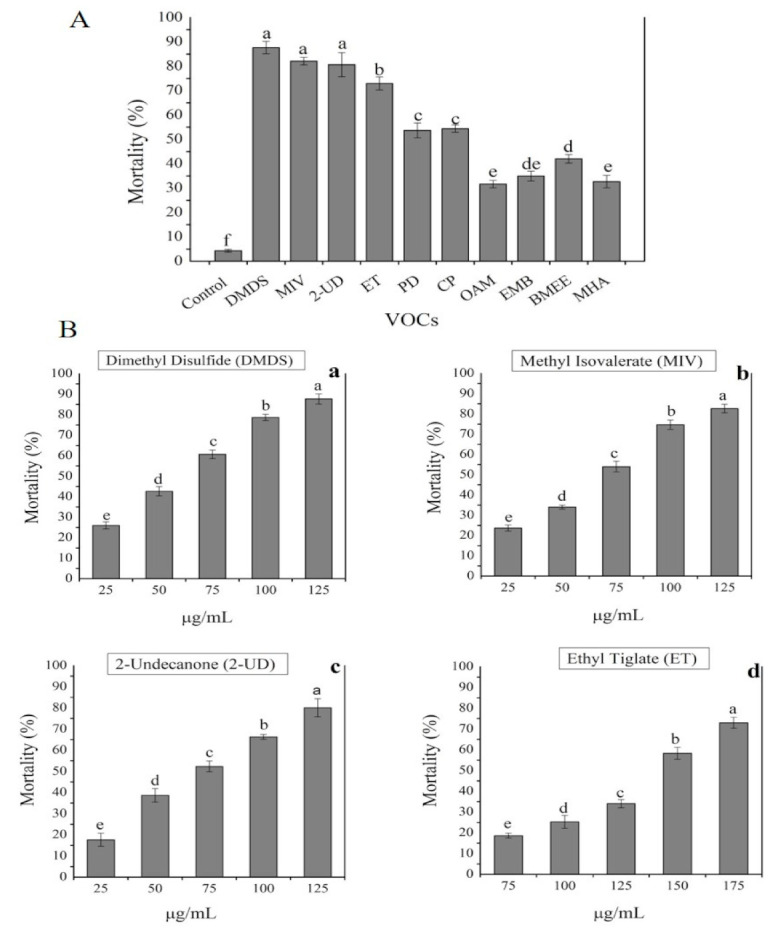
Nematicidal effect of pure VOCs with different concentrations against *M. incognita*. (**A**) The mortality rate of each pure VOC compared with control. (**B**) The killing effect of four strong nematicidal VOCs (**a**–**d**) with different concentrations. The error bars represent the mean standard deviation of each treatment repeated three times with three replicates. The small letters above the columns indicate significant difference. The significance differences among treatments were calculated by Tukey’s HSD test at *p* ≤ 0.05.

The possible pure VOCs identified through GC-MS analysis listed in Table 1 were purchased from Sigma-Aldrich company supplier in Shanghai, China and were screened individually for their nematicidal activity with different concentrations. The three VOCs, i.e., dimethyl disulfide (DMDS), methyl isovalerate (MIV), and 2-undecanone (2-UD), were found to have strong nematicidal activity with a mortality rate of 87%, 83%, and 80%, respectively, followed by ethyl tiglate (70%), and cyclopentene (67%) mortality rates. Moreover, the above three VOCs up to 200 µg/mL concentration resulted in (100%) mortality. The other VOCs, i.e., butanoic acid, 3-methyl, ethyl ester (BMEE), ethyl-2-methyl butyrate (EMB), 2-methylheptonic acid (MHA), octanoic acid 2-methyl (OAM), and pentadecane (PD) showed 50%, 45%, 40%, 43%, and 55% mortality rates, respectively, as shown in Figure 4.

### 2.5. Induction of ROS in Meloidogyne incognita Exposed to Pure Volatiles

**Figure 5:**
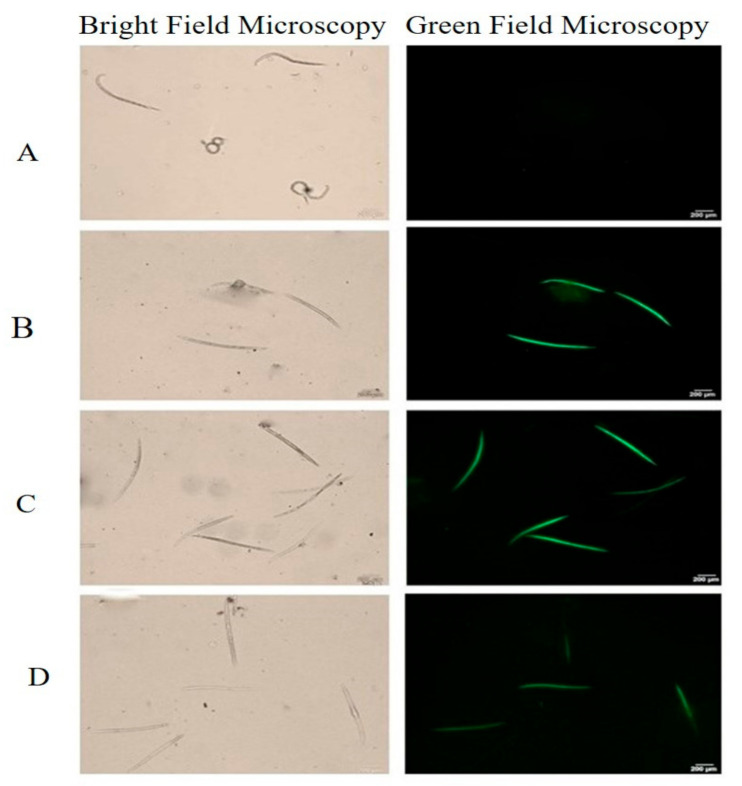
ROS induction by pure volatiles. (**B**) Methyl isovalerate (MIV), (**C**) dimethyl disulfide (DMDS), and (**D**) 2-undecanone (2-UD) in nematodes compared with (**A**) as control unexposed to volatiles having no ROS production. The higher level of green fluorescence indicates the high level of ROS induction in nematodes exposed to the selected volatiles. Scale bar = 200 μm.

Exposure to toxic compounds that elicit ROS production resulted in abnormal cell homeostasis and finally death [36,37]. The current results show that MIV, 2-undecanone (2-UD) and dimethyl disulfide (DMDS) with strong nematicidal activity-induced ROS in nematodes. The induction of ROS in the nematodes exposed to the selected VOCs led to severe oxidative stress that resulted in death. Furthermore, the high-level green fluorescence shows high ROS induction in the nematodes pre-exposed to volatiles. The MIV and DMDS were observed to induce severe oxidative stress followed by 2-UD, as shown in Figure 5.

### 2.6. The Pure VOCs Have the Potential to Enhance In Vitro Plant Growth in Tomato

**Figure 6:**
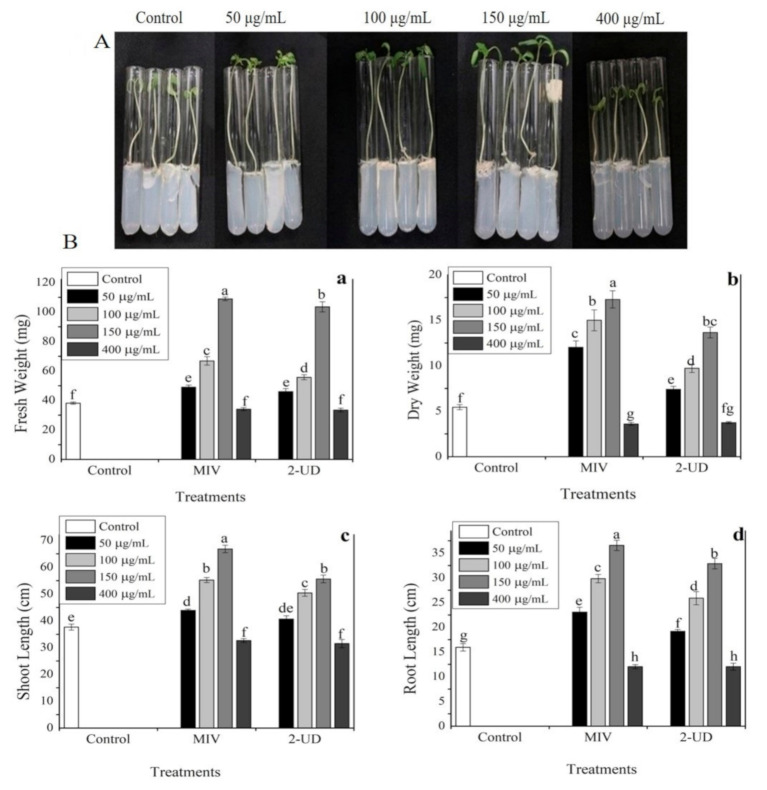
The exposure of tomato seedlings to pure VOCs, i.e., methyl isovalerate (MIV) and 2-undecaone (2-UD), at different concentrations of (50 µg/mL, 100 µg/mL, 150 µg/mL, and 400 µg/mL) grown in test tubes kept in an airtight jar for 12 days at 25 °C under 12/12 h dark photoperiod. (**A**) Visual presentation of methyl isovalerate (MIV) effect on tomato plant growth. (**B**) Growth parameters recorded: (**a**) fresh weight (**b**) dry weight, (**c**) shoot length, and (**d**) root length. Error bar represents the mean standard deviation of each treatment. The small letters above the columns indicate significant difference. The significant difference between the treatments was determined through Tukey’s HSD test at *p* ≤ 0.05.

Dimethyl disulfide (DMDS) was previously reported to have the potential to promote plant growth and to induce systemic resistance against plant pathogens. Therefore, the other pure volatiles listed in Table 1 were focused on to evaluate their potential for enhancing plant growth in the current study. Among pure VOCs, methyl isovalerate (MIV) and 2-undecanone (2-UD) were observed to have a high growth-promoting effect, followed by cyclopentene (CP) and ethyl tiglate (ET). The result showed that two compounds, i.e., methyl isovalerate and 2-undecanone (2-UD) with concentration up to 150 µg/mL, significantly enhanced tomato plant growth in terms of shoot length (38%), root length (31%), fresh weight (40%), and dry weight (2%) as compared with DMSO as the control, followed by 2-UD with enhancement in shoot length (37%), root length (30%), fresh weight (35%), and dry weight (25%), respectively. Furthermore, a slight negative impact on plant growth was observed when VOCs were used at a high concentration of 400 µg/mL, as shown in Figure 6.

### 2.7. Plant Growth Promotion by Methyl Isovalerate (MIV) and 2-Undecaone (2-UD) in the Greenhouse Experiment

**Figure 7:**
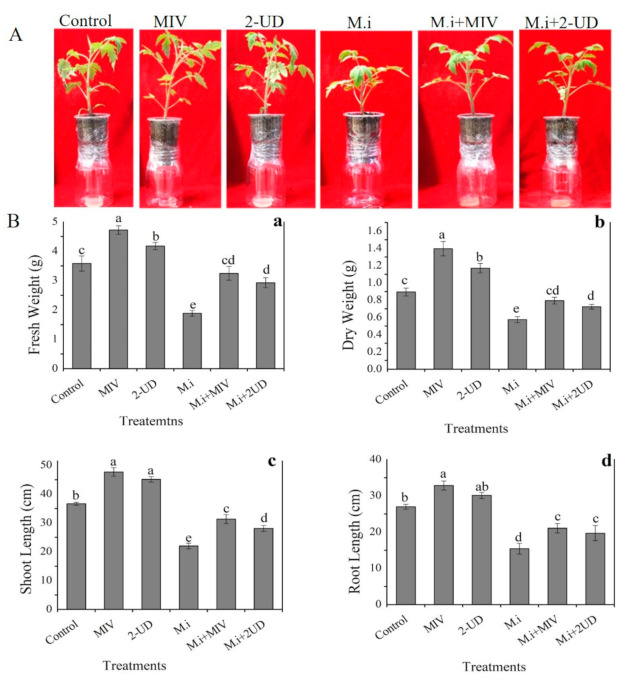
The effect of MIV and 2-UD *M. incognita* (M.i)-infested tomato plant in a pot experiment. The healthy plants having no M.i were used as control. (**A**) Visual presentation of the effect MIV and 2-UD on tomato plant growth. (**B**) Graphical display of the above VOCs on growth promotion by calculating different parameters: (**a**) fresh weight, (**b**) dry weight, (**c**) shoot length, (**d**) root length. The error bar represents the mean standard deviation of each treatment repeated three times with three replicates. The small letters above the columns indicate significant difference. The significant difference between different treatments was calculated through Tukey’s HSD test at *p* ≤ 0.05.

**Figure 8:**
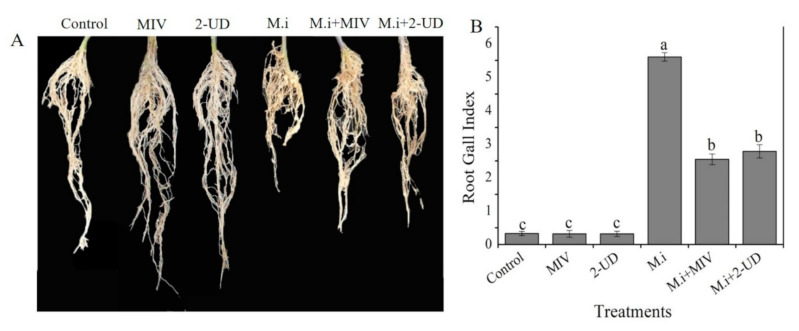
Reduction in the number of root galls in *M. incognita* (M.i)-infested plants after being exposed to pure volatiles, i.e., methyl isovalerate (MIV) and 2-undecanone (2-UD), up to 40 days was observed visually in pot experiments. (**A**) The effect of volatiles on root length and reduction in root gall in an infested plant (M.i) after being exposed to volatiles. (**B**) Graphical information of root gall reduction in the term of root gall index in infested plant pre-exposed to MIV and 2-UD. The error bar represents the mean standard deviation of each treatment repeated three times with three replicates. The small letters above the columns indicate significant difference. The significant difference between different treatments was calculated through Tukey’s HSD test at *p* ≤ 0.05.

In the current study, the effect of pure volatiles, i.e., methyl isovalerate (MIV) and 2-undecanone (2-UD), with strong nematicidal activity was studied for plant growth promotion and induced systemic resistance against *M. incognita* at a broader level in tomato plants in an experiment in greenhouse designed according to the previous protocol with some modifications [14,38,39]. Four-leaf stage tomato seedlings of equal size were transplanted into each pot attached to a jar having pure methyl isovalerate (MIV) and 2-undecanone (2-UD) at different concentrations up to 150 µg/mL. Tomato plants in plastic pots with small holes (4–6) at the bottom were inoculated with *M. incognita* (500 J2 Juveniles) to induce root-knot disease. The small holes in the pot allowed the VOCs to come in contact with the infested roots for the purpose of studying the effect of volatiles on plant growth promotion and disease reduction. Analysis of data recorded after 40 days showed a significant increase in plant growth in terms of shoot length, root length, dry weight, and fresh weight and alleviation of root-knot disease by a reduction in the number of root galls present on the infested roots as compared with water or DMSO as control. The data revealed an increase in all growth parameters; shoot length (37%), root length (33%), fresh weight (40%), and dry weight (28%) was noticed for methyl isovalerate (MIV), and shoot length (34%), root length (32%), fresh weight (37%), and dry weight (27%) was noticed for 2-Undecanone, as shown in Figure 7. Furthermore, an alleviation in disease severity was observed by calculating the number of root galls in terms of root gall index on infested roots. The number of root galls in the infested roots was significantly reduced after exposure to MIV followed by 2-UD compared with control, as shown in Figure 8. According to the data obtained from the experiments, we can state that enhancement in plant growth and reduction in the severity of root-knot disease was noted after exposure to MIV and 2-UD in pot experiments.

### 2.8. Volatiles Produced by GBSC56 Enhance Antioxidant Enzymes Activity in M. incognita-Infested Tomato Plants

**Figure 9:**
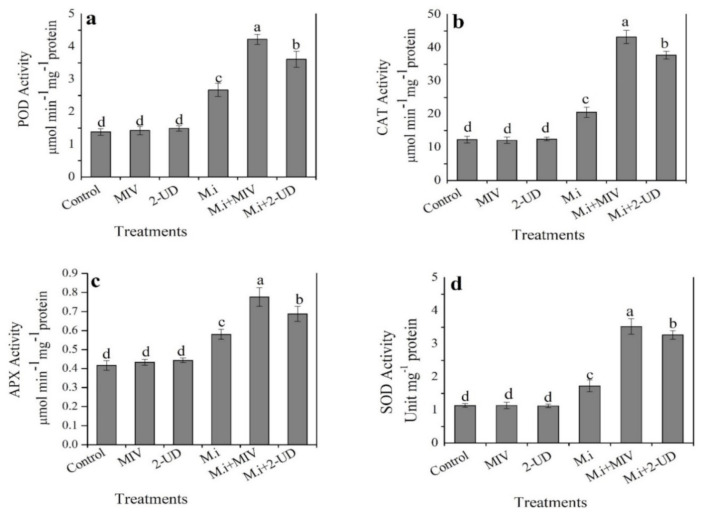
The effect of pure volatiles, i.e., methyl isovalerate (MIV) and 2-undecanone (2-UDE), on antioxidant enzymes. (**a**–**d**) Activity in *M. incognita-*infested and non-infested tomato plant roots. The root samples of different treatments were taken at 4 dpi to obtain enzyme extract. Error bar represents the mean standard deviation of three repeats with three replicates for each treatment. The small letters above the columns indicate significant difference. The significant difference among the treatments was calculated through Tukey’s HSD test at *p* ≤ 0.05.

The activity of antioxidant enzymes (SOD, CAT, POD, and APX) was evaluated in tomato plants infested with *M. incognita* after being exposed to pure volatiles, i.e., methyl isovalerate (MIV) and 2-undeanone (2-UD). The results show that the activity of all enzymes was increased in *M. incognita*-infested plants as compared with non-infested plants taken as control. The overall data revealed that a significant stimulation was observed in the activity of all enzymes in *M. incognita*-infested plants after exposure to MIV followed by 2-UD, as shown in Figure 9.

### 2.9. Expression Analysis of Defense-Related and PGP Genes in Meloidogyne incognita-Infested Tomato Root Pre-Exposed to Volatiles

**Figure 10:**
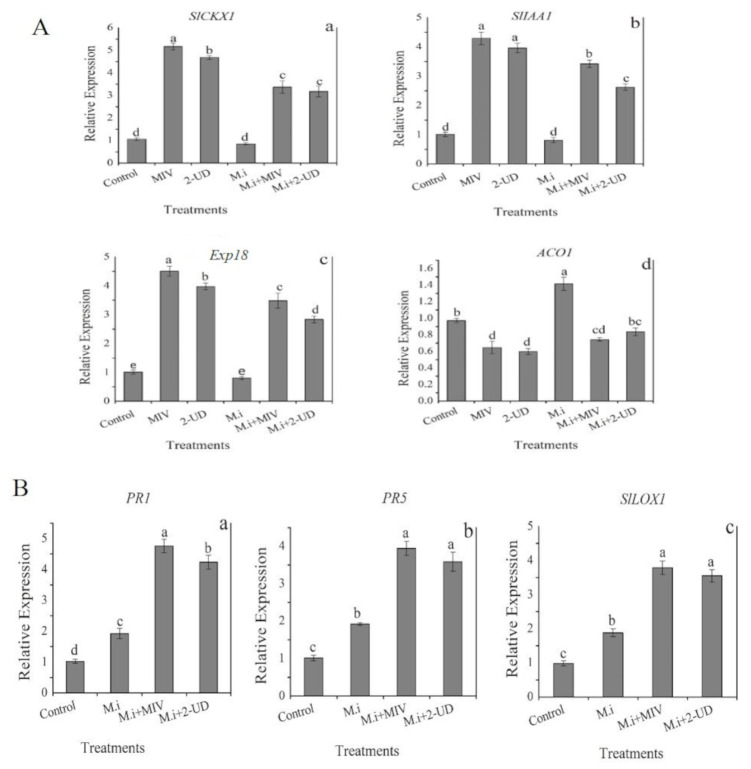
(**A**) Expression analysis of growth-promoting genes (**a**) *SlCKXI*, (**b**) *SlIAA1,* (**c**) *Exp18*, and (**d**) *ACO1*) in *M. incognita* (M. i)-infested tomato plant at 5 dpi pre-exposed to pure volatiles, i.e., MIV and 2-UD. (**B**) The expression profile of defense-related genes (**a**) *PR1*, (**b**) *PR5*, and (**c**) *SlLOX1* in *M. incognita*-infested plants pre-exposed to MIV and 2-UD as compared with healthy control. The error bar indicates the mean standard deviation of each treatment repeated three times with three replicates. The different small letters above the columns indicates significant difference according to Tukey’s HSD test at *p* ≤ 0.05.

To investigate whether the pure volatiles, i.e., methyl isovalerate (MIV) and 2-undecanone, can regulate the expression of genes involved in plant defense and growth promotion, we examined the transcriptional regulation of seven genes, i.e., four growth-promoting genes (*SlCK1*, *SlIAA1*, *ACO1*, and *EXP18*) and three defense-related genes (*PR1*, *PR5*, and *SlLOX1)*, through quantitative PCR (qRT-PCR) analysis. *M. incognita*-inoculated tomato root samples exposed to VOCs were taken at 5 dpi. The total RNA was extracted, and cDNA was synthesized from polyadenylated mRNA. Furthermore, qPCR was performed for the selected genes with different gene-specific primers (Table S1). The expression of genes (*SlCK1*, *SlIAA1*, and *EXP18*) was observed to be enhanced while downregulation was seen for the *ACO1* gene both in infested and non-infested tomato root after exposure to MIV and 2-UD as compared with water control. Moreover, upregulation for *SCLK1* and *Exp18* gene expression was higher in tomato roots exposed to MIV followed by 2-UD. The expression of defense-related genes (*PR1*, *PR5*, and *SlLOX1*) was observed be to stimulated by MIV and 2-UD. The highest expression for the *PR1*gene was observed in infested roots exposed to MIV followed by *SlLOX1* and *PR5*, as shown in Figure 10. The overall results from the experiment reveal that defense-related and plant growth-promoting genes in *M. incoginta*-infested roots are highly regulated by MIV and 2-UD.

## 3. Discussion

Plant-parasitic nematodes are devastating to crops and are becoming a major threat to crop productivity [1]. Although chemical nematicidal agents offer remedial solutions and protection from huge losses, their detrimental effects—such as to human health and environmental quality—discourage their widespread use [9,40]. Hence the application of microbial VOCs has shown a significant antagonistic effect against different plant nematodes, stimulating growth and triggering induced systemic resistance (ISR) in infested plants [20,41], and they are considered a safe and environmentally friendly method [13]. In the light of these characteristics, the scientific community has shown huge interest in finding novel microbial VOCs for controlling plant diseases [42]. Existing evidence proves that bacterial VOCs such *Bacillus* strains produce nematicidal VOCs that stimulate ISR against *M. incognita* and promote plant growth [43,44].

The study was aimed to explore GBSC56-VOC’s nematicidal activity, along with their potential to stimulate plant growth and to stimulate ISR against *M. incognita*, both in vitro and in planta experiments. The current findings show that GBSC56-VOCs have strong nematicidal activity against *M. incognita*, with a mortality rate of 90% as compared with control. The GBS56-VOCs were further identified by GC-MS analysis to possess the most active volatiles against the nematode. Among 10 pure VOCs identified, dimethyl disulfide (DMDS), methyl isovalerate (MIV) and 2-undecanone (2-UD) were observed to have strong nematicidal activity. The 2-UD and DMDS were previously reported to have nematicidal activity against *M. incognita* [45], but to the best of our knowledge, MIV is the newest volatile noticed for the first time in the current study to have strong nematicidal activity against the nematode. These VOCs were further found to induce excessive ROS production in *M. incognita*, also reported for the first time in the study. Previously, 1, 4-naphthoquinone was reported to stimulate ROS induction that caused rapid death of *C. elegans* [37]. The oxidative stress could cause cell death by inducing damage to proteins, lipids, and DNA [46]. Hence, we assumed that the VOCs (DMDS, MIV, and 2-UD) in the current study could also cause severe oxidative stress and negatively affect the *M. incognita* antioxidant enzymes activity that results in high ROS production and internal damage to nematodes. These findings further suggest that research should study the detailed mechanism of induced damage in *M. incognita* by the selected volatiles through negative regulation of antioxidant enzymes activity in nematodes.

The nematicidal VOCs produced by GBSC56 were also studied for their potential to promote growth and to stimulate ISR in *M. incognita-*infested tomato plants through in vitro and in planta experiments. In the previous study, most researchers used the volatiles to promote plant growth in healthy plants, but the mechanism of growth promotion in infected plants exposed to volatiles was still at the preliminary stages [47]. The findings of Tahir et al. (2017) demonstrated the role of SYST2-VOCs in plant growth promotion. Furthermore, the BCT9 strain was also reported to produced volatiles, such as 3-hydroxy-2-butanone, 2,3-butanediol, 2-nonanone, 2-tridecanone, and 2-pentadecanone, with the potential to stimulate plant growth [48]. The VOCs (MIV and 2-UD) with strong nematicidal activity in the current study are reported here for the first time to stimulate growth in *M. incoginta*-infested tomato plants. The study provides new insight into the mechanism of volatiles (MIV, 2-UD) by regulating the key genes involved in plant growth promotion in infested plants. The VOCs were also observed to induced ISR that results in root galls reduction in infested plants. The number of root galls was significantly reduced in infested tomato exposed to pure volatile (MIV and 2-UD), indicating that the VOCs can reduce *M. incognita* infectivity and reproduction. In addition, these results strongly support the idea that bacterial VOCs may reduce the movement of nematodes toward roots, negatively affecting their potential to cause severe root gall formation and egg production in tomato roots [49].

The antioxidant enzymatic system plays a significant role in plant resistance against different pathogens, minimizing the adverse effect of ROS induced after infection [50]. Previously, the volatiles produced by *Bacillus subtilis* CF-3 reduced the disease progression of *Colletotrichum gloeosporioides* on the harvested lychee fruits and positively regulated the antioxidant enzymes activity of fruits to minimize the adverse effect of oxidative stress induced after infection [51]. Studies conducted in the past also reported an upsurge in the antioxidant enzymes activity of plants infected with phytopathogenic bacteria [52]. However, little is known about the nematicidal volatiles that can regulate antioxidant enzyme activity in tomato roots infested with *M. incognita*. In the current study we noticed for the first time that the nematicidal volatiles (MIV and 2-UD) stimulate antioxidant enzyme activity in *M. incognita*-infested tomato roots, which might reduce the adverse effect of oxidative stress induced after infection.

Bacterial volatiles can promote plant growth and stimulate ISR against various plant pathogens by regulating the genes involved in different signaling pathways [53]. For example, exposure to SYST2-VOCs has been reported to upregulate the expression of *SlCKX1, SlAA1*, and *Exp18* genes in tomato roots [14]. The cytokinin pathway in growth regulation stimulated by bacterial VOCs is considered to be more important because of the crucial role of cytokinin reported earlier in root development [54]. Furthermore, the upsurge in the expression of expansin genes such as *EXP4*, *EXP5*, *EXPB1*, and *EXPB3* has been reported in *Arabidopsis* after exposure to volatiles [20]. MIV and 2-UD were also noticed in the present study to positively regulate growth-promoting genes (such as *SlCKX1*, which is involved in the cytokinin production pathway; *SlIAA1*, which is involved in auxin synthesis pathway; and *exp18*, which is involved in expansin production) for the purpose of developing tomato roots. Moreover, the genes involved in ethylene synthesis (*ACO1, ACO2, ACS12, ACS4*, and *SAM-2)* were reported to be negatively regulated by volatiles in plants under stress condition [55]. In the current study, the *ACO-1* gene responsible for the final step in ethylene biosynthesis pathways was also noticed to be highly downregulated in *M. incognita*-infested tomato roots pre-exposed to MIV and 2-UD, supporting the previous findings. Moreover, the stimulation of ISR in infected plants pre-exposed to volatiles without any physical contact (infected plants exposed to volatiles from a distance in a close container rather than adding the VOCs directly in the soil) shows their important role in plant disease resistance against different phytopathogens [56]. Previous findings mentioned that microbial VOCs have the potential to trigger ISR to protect the plant against plant pathogens through coordinated signaling pathways and defense-linked genes that alleviate the risk of plant diseases [57,58]. The current study for the first time reports strong nematicidal volatiles (MIV and 2-UD) with the potential to trigger ISR against *M. incognita* in infested tomato through positive regulation of defense-related genes (*PR1*, *PR5*, and *SlLOX1*), achieving better management of root-knot disease.

## 4. Material and Methods

### 4.1. Bacterial Culture and In Vitro Tomato Seedlings Growth Conditions

The *Bacillus* strain GBSC56 isolated previously in our Laboratory of Biocontrol and Bacterial Molecular Biology (Nanjing Agriculture University, Nanjing, China) was used in the present study. The fresh culture was obtained by growing the strain on (LB) Luria–Bertani medium at 37 °C, and culture was stocked in LB broth supplemented with 30% glycerol at −80 °C. The seeds of the tomato were first washed with 70% ethanol, then soaked in sodium hypochlorite solution (50%) for 15 min, followed by rinsing with distilled water (ddH_2_O) 4–5 times. For in vitro growth assay, the sterilized seeds were placed in test tubes filled with 0.5× Murashige and Skoog (MS) medium (pH 5.7) [59] with 1.5 g sucrose and agar (0.08%) as a supplementary source.

### 4.2. Collection of Meloidogyne incognita Eggs and Second-Stage Juveniles

The original culture of *M. incognita* was provided by Professor Wang Xuan (Laboratory of Plant nematology, Nanjing Agricultural University, Jiangsu, China). Tomato plants infected with *M. incognita* were grown in a greenhouse with a controlled environment under 23–28 °C and relative humidity of 40–60%. After 40 days post infestation (dpi), the infested roots were taken and washed with distilled water to remove the soil particles, followed by collecting the egg masses with a dissecting needle [60]. Surface sterilization of the egg masses was then carried out with 1% NaOCl (sodium hypochlorite) for 3 min and then washed several times with distilled water [61]. Finally, from eggs, the second-stage (J2) juveniles of *M. incognita* were obtained using the Baermann funnel method [62].

### 4.3. Evaluation of GBSC56-VOCs for Their Nematicidal Activity and ROS Induction in Meloidogyne incognita

The two-compartments Petri plate method was used to determine the nematicidal activity of GBSC56-VOCs, as described by [63] with some modifications. Briefly, the fresh culture of GBSC56 was grown on LB medium for 24 h at 28 °C in one compartment of the partition plate having 2% water agar (WA) in the second portion. *M. incognita* (200 J2 juveniles) were then added after 24 h to the second compartment with WA. Furthermore, the Petri plates were then firmly sealed with parafilm for the purpose of stopping the escape of volatiles. After incubation at 25 °C for 48 h, mobile and immobile nematodes in WA compartments were viewed through the light microscope. Immobile nematodes were considered as dead unable if unable to regain movement after added to water and touched with a needle under a microscope. The non-inoculated LB medium was kept as a control treatment. The experiment was repeated three times.

### 4.4. GC-MS Profiling of VOCs Produced by GBSC56

The 20 µL of GBSC56 cell suspension was grown for 4 days on 30 mL of LB agar medium placed at 28 °C in a 100 mL vial. The non-inoculated LB agar medium in the vial was kept as control. Four days later, the VOCs produced by the strain were collected with 2 cm microextraction solid-phase fiber (divinylbenzene/carboxen/PDMS (DCP, 50/30 µm) (Supelco, Bellefonta, PA, USA) inserted into the vial through the headspace at 50 °C for 30 min containing bacteria. The instrument (Bruker 450-GC gas chromatography) connected with (Bruker 320-MS mass spectrometer) was used for gas chromatography-mass spectrometry (GC-MS) analysis according to the given protocol [60]. The instrument used helium gas as the carrier with a flow rate of 1 mL min^−1^. The SPME fibers were desorbed for 5 min at 220 °C, followed by operating the GC-MS for 50 min. First, the column (BR-5ms, Cat. # BR-86377 from USA) temperature was maintained for 2 min at 40 °C, after that increased at 4 °C/min rate to 180 °C, and then reached to 240 °C with the rate of 5 °C/min held for 6 min. The mass spectrometer was operated in an electron ionization mode of 70 eV with a source temperature of 220 °C and frequently scanned 50 to 500 m/z. The identification of volatile compounds was confirmed by comparing their spectra and retention time with data from the NIST/EPA/NIH mass spectrum library. Moreover, similar volatiles in the control with high peak area, i.e., ≥1%, were excluded from the volatiles produced by GBSC56 strain according to the previous study [14]. The possible volatiles identified through GC-MS analysis were bought from Sigma-Aldrich company, Shanghai China. The individual compounds were then studied for nematicidal activity against *M. incognita*, along with growth-promoting potential and stimulation of induced systemic resistance in the tomato plant.

### 4.5. In Vitro Nematicidal Activity of Pure VOCs Against Meloidogyne incognita

The pure volatiles identified through GC-MS analysis were evaluated for nematicidal activity *M. incognita* with different concentrations in a 96-well plate experiment. The compounds were diluted in ethanol, then added to water having polysorbate surfactant Tween 20 according to the given protocol [23,38,45,49] with some modifications. To find the nematicidal activity of each pure VOC with different concentrations, a total volume of 120 μL per well was added to 96-well plates. Moreover, *M. incognita* J2 form (About 30 nematodes/well) was transferred to each well already filled with pure VOCs. Wells containing solvent were kept as control. Each treatment was performed thrice with three replicates. The plates were then covered with plastic lids and placed in an incubator in dark for 24 h 25 °C. After 24 h of exposure, observations were recorded under an inverted microscope. Still nematodes were teased and their movement was observed within 2 s, and they were counted dead if the movement was not regained. With the help of Schneider–Orelli formula [64], the error caused by the natural death of nematodes was corrected by comparing the treated samples with negative control treatment.

### 4.6. Reactive Oxygen Species (ROS) Assay

GBSC56-VOCs or individual pure volatiles-induced ROS production in *M. incognita* was detected as described by Kim et al. [3] with some modifications. Briefly, nematodes were exposed to pure volatiles, followed by incubating for 48 h at 25 °C. Nematodes after exposure to VOCs were collected in 1.5 mL Eppendorf tubes through centrifugation at 700 rpm at room temperature. The collected nematodes were then rinsed thrice with distilled water treated with ROS detection chemical named dichloro-dihydro-fluorescein diacetate (DCFH-DA) (JianCheng Bioengineering, Nanjing, China) mixed with 10 mM sodium phosphate buffer (pH 7.4) [65]. The dead nematodes containing ROS and stained by the dye gave green fluorescence and were observed with a microscope (Olympus1 × 71) programmed with Image-Pro express software v.6.2 (Olympus, Tokyo, Japan).

### 4.7. Volatiles with Potential to Stimulate Plant Growth and Induced Systemic Resistance

The pure VOCs with nematicidal activity were further evaluated for plant growth promotion and were induced in tissue culture jar and pot experiments according to the protocol given by [38,66] with some modifications. Briefly, four test tubes containing 0.5× MS medium were transferred to an autoclaved tissue culture jar (12 cm × 7 cm). Petri plates with LB medium or empty (4 cm × 1.5 cm) were placed at the bottom of the jar. Sterilized tomato seeds per tube containing MS media (2/3 filled) were placed in a tissue culture jar closed firmly with a lid to avoid the escape of volatiles produced by 20 µL GBSC56 fresh culture (10^7^ CFU/mL) grown on the small plate containing LB medium. Furthermore, the jars were then incubated at 25 °C for 12 days under a photoperiod of 12 h light/12 h dark. The same method was used to analyze the potential of each pure VOC, with different concentrations diluted in dimethyl sulphoxide (DMSO) as a solvent. The lids on the jars were firmly closed to avoid the escape of volatiles from the jar. For the pot experiments, the pure VOCs with different concentrations were added to small Petri plates and put in a tissue culture jar (12 cm × 10 cm) as mentioned in the protocol [16]. Furthermore, equal size four-leaf stage tomato seedlings were transferred to plastic pots (6 cm × 3 cm) with small holes (4–5) at the bottom and filled with soil (proper amounts of sand, clay, and organic matter). The plastic glasses with tomato seedlings were then fixed on the tissue culture jars containing VOCs and sealed with parafilm firmly to stop the escape of volatiles. The *M. incognita* J2 juveniles (500 nematodes/pot) were added to induce root-knot disease. The plastic holes enable the roots of tomato plants to make a connection with volatiles at the bottom of the jar. The plastic pots with tomato seedlings fixed on jar inoculated with *M. incoginta* or un-inoculated used as control were kept in the controlled environment of a greenhouse at 25 °C under a 12 h light/12 h dark photoperiod for 40 days. Parameters such as shoot length, root length, fresh weight, dry weight, and root gall index were calculated to study the effect of GBSC56 volatiles on growth promotion and on reduction of root-knot disease in tomato plants.

### 4.8. Antioxidant Enzymes Activity Assay

The enzymatic activity of four antioxidant enzymes such as peroxidase (POD), superoxide dismutase (SOD), catalase (CAT), and ascorbate-dependent peroxidase (APX) was determined spectrophotometrically at early stage at 4 days post-inoculation (dpi) of *M. incognita*-infested roots exposed to MIV and 2-UD according to the mentioned protocol [67]. Briefly, 0.3 g fresh frozen root samples were ground in the phosphate buffer solution with pH 7.8 in an ice bath, followed by centrifugation at 4 °C with a maximum speed of 1200× *g* for 30 min. The supernatant was used as an enzyme extract. The absorbance was determined at 470 nm for POD, 560 nm for SOD, 240 nm for CAT, and 290 nm for APX, taking ddH2O as a reference.

### 4.9. Expression Profiling of Genes Involved in Growth Stimulation and Defense in Tomato

The expression profile of genes involved in growth promotion and defense of tomato plants against *M. incognita* were studied after exposure to pure VOCs, i.e., methyl isovalerate (MIV) or 2-undecanone (2-UD), in a pot experiment. For RNA extraction, the roots samples were harvested at 5 days post-inoculation (dpi) from *M. incognita*-infested or non-infested tomato for selected gene expression analysis. The total RNA extraction was carried out with the given protocol with a bacterial RNA extraction kit (OMEGA Bio-tek, Inc. Norcross, GA, USA), followed by quantification through Nanodrop (Nanodrop 1000, Thermo Scientific, Wilmington, DE, USA). Moreover, the cDNA construction was performed according to the protocol of 5× All-In-One RT Master Mix (with AccuRT Genomic DNA Removal Kit) by Applied Biological Materials Inc. (abm^®^, Beijing). The Step One Real-Time PCR System (Applied Biosystems, Foster City, CA, USA) was used for RT-qPCR analysis. The SYBR Green (Takara Bio, Beijing, China), which emits fluorescence to cDNA, was used as a detector. The value of the fluorescence was noted at the threshold cycle (C). Primers were designed through the Primer Quest tool for selected genes listed in Table S1. The housekeeping gene actin previously described in tomato as endogenous control was used in the present study [68]. The 20 μL qPCR reaction mixture contained 10 µL 2X SYBR premix Ex Taq (Takara Bio, Beijing, China) (Til RNaseH Plus) with Rox as a reference dye, 0.4 µL forward and reverse primers (20 nmol), 2 µL cDNA (100 ng), and 6.8 µL ddH_2_O. The PCR machine was programmed using the following steps: i.e., initial denaturation at 95 °C for 30 s, including 40 cycles of 95 °C for 5 s, and 34 s at 60 °C. Finally, relative quantification was performed based on the comparative C method of 2−ΔΔCT as described by [69].

### 4.10. Statistical Analysis

All experiments were conducted in a completely randomized design. Experimental data were subjected to statistical analysis using Statistics 8.1 software (analytical software, USA) [70]. Means were separated using Tukey’s HSD test at *p* ≤ 0.05 after ANOVA.

## 5. Conclusions

*Bacillus* sp. strain GBSC56 obtained from the Tibet region of China showed high nematicidal activity against *M. incognita.* The GBCS56-VOCs or pure chemical volatiles, i.e., methyl isovalerate (MIV), 2-undecanone (2-UD), and dimethyl disulfide (DMDS), induced severe oxidative stress in nematodes and resulted in their rapid death. Moreover, the study provides new insight into the mechanism by which nematicidal volatiles regulate antioxidant enzymes activity, the key genes involved in plant growth promotion, and the defense mechanism *M. incognita*-infested plants use to efficiently manage root-knot disease.

## Figures and Tables

**Table 1:** The possible volatile organic compounds (VOCs) produced by GBSC56 were purchased from Sigma-Aldrich company supplier, Shanghai, China.

S. No	Possible Compounds	Case No	Molecular Weight	RT	Prob%	Molecular Formula
1	Methyl Isovalerate	556-24-1	116	4.75	65.2	C_6_H_12_O_2_
2	Ethyl-2-methyl butyrate	452-79-1	130	11.73	28.7	C_7_H_14_O_2_
3	2-undecanone	112-12-9	170	21.70	45.4	C_11_H_22_O
4	Butanoic acid,3-methyl, ethyl ester	452-79-1	130	7.17	76.1	C_7_H_14_O_2_
5	Octanoic acid 2-methyl	004-93-1	158	12.72	42.1	C_9_H_18_O_2_
6	Disulfide, Dimethyl	624.92.0	97	3.45	68.2	C_2_H_6_S_2_
7	2-Methylhepatonic acid	188-02-9	144	11.82	21.24	C_8_H_16_O_2_
8	Pentadecane	629-62-9	222	28.43	35.7	C_15_H_22_
9	Ethyl Tiglate	837-78-5	128	9.53	43.3	C_7_H_12_O_2_
10	Cyclopentene	142-29-0	68	1.52	12.1	C_5_H_8_

## Data Availability

Not applicable

## Supplementary Materials

The following are available online at https://www.mdpi.com/article/10.3390/ijms22095049/s1, Table S1: Primers used in the study.

## References

[1] Li J., Zou C., Xu J., Ji X., Niu X., Yang J., Huang X., Zhang K.-Q. (2015). Molecular mechanisms of nematode-nematophagous microbe interactions: Basis for biological control of plant-parasitic nematodes. Annu. Rev. Phytopathol..

[2] Kyndt T., Fernandez D., Gheysen G. (2014). Plant-Parasitic Nematode Infections in Rice: Molecular and Cellular Insights. Annu. Rev. Phytopathol..

[3] Kim T.Y., Jang J.Y., Jeon S.J., Lee H.W., Bae C.H., Yeo J.H., Lee H.B., Kim I.S., Park H.W., Kim J.C. (2016). Nematicidal activity of kojic acid produced by *Aspergillus oryzae* against *Meloidogyne incognita*. J. Microbiol. Biotechnol..

[4] Nicol J.M., Turner S.J., Coyne D.L., den Nijs L., Hockland S., Maafi Z.T. (2011). Current nematode threats to world agriculture. Genomics and Molecular Genetics of Plant-Nematode Interactions.

[5] Vos C., Schouteden N., van Tuinen D., Chatagnier O., Elsen A., De Waele D., Panis B., Gianinazzi-Pearson V. (2013). Mycorrhiza-induced resistance against the root-knot nematode *Meloidogyne incognita* involves priming of defense gene responses in tomato. Soil Biol. Biochem..

[6] Trudgill D.L., Blok V.C. (2001). Apomictic, polyphagous root-knot nematodes: Exceptionally Successful and Damaging Biotrophic Root Pathogens. Annu. Rev. Phytopathol..

[7] Gravato-Nobre M.J., McClure M.A., Dolan L., Calder G., Davies K.G., Mulligan B., Evans K., Von Mende N. (1999). *Meloidogyne incognita* surface antigen epitopes in infected *Arabidopsis* roots. J. Nematol..

[8] Boland G.J., Hall R. (1994). Index of plant hosts of *Sclerotinia sclerotiorum*. Can. J. Plant Pathol..

[9] Riga E. (2011). The effects of *Brassica* green manures on plant parasitic and free-living nematodes used in combination with reduced rates of synthetic nematicides. J. Nematol..

[10] Lu H., Xu S., Zhang W., Xu C., Li B., Zhang D., Mu W., Liu F. (2017). Nematicidal activity of trans-2-hexenal against southern root-knot nematode (*Meloidogyne incognita*) on tomato plants. J. Agric. Food Chem..

[11] Li N., Pan F.J., Han X.Z., Zhang B. (2016). Development of soil food web of microbes and nematodes under different agricultural practices during the early stage of pedogenesis of a Mollisol. Soil Biol. Biochem..

[12] Song G.C., Ryu C.M. (2013). Two volatile organic compounds trigger plant self-defense against a bacterial pathogen and a sucking insect in cucumber under open field conditions. Int. J. Mol. Sci..

[13] Tahir H.A.S., Gu Q., Wu H., Raza W., Safdar A., Huang Z., Rajer F.U., Gao X. (2017). Effect of volatile compounds produced by *Ralstonia solanacearum* on plant growth promoting and systemic resistance inducing potential of *Bacillus* volatiles. BMC Plant Biol..

[14] Tahir H.A.S.S., Gu Q., Wu H., Raza W., Hanif A., Wu L., Colman M.V., Gao X. (2017). Plant growth promotion by volatile organic compounds produced by *Bacillus subtilis* SYST2. Front. Microbiol..

[15] Zou C., Li Z., Yu D. (2010). *Bacillus megaterium* strain XTBG34 promotes plant growth by producing 2-pentylfuran. J. Microbiol..

[16] Park Y.-S., Dutta S., Ann M., Raaijmakers J.M., Park K. (2015). Promotion of plant growth by *Pseudomonas fluorescens* strain SS101 via novel volatile organic compounds. Biochem. Biophys. Res. Commun..

[17] Das A., Lee S.-H., Hyun T.K., Kim S.-W., Kim J.-Y. (2013). Plant volatiles as method of communication. Plant Biotechnol. Rep..

[18] Cutler S.R., Rodriguez P.L., Finkelstein R.R., Abrams S.R. (2010). Abscisic acid: Emergence of a core signaling network. Annu. Rev. Plant Biol..

[19] Ping L., Boland W. (2004). Signals from the underground: Bacterial volatiles promote growth in *Arabidopsis*. Trends Plant Sci..

[20] Zhang H., Kim M.S., Krishnamachari V., Payton P., Sun Y., Grimson M., Farag M.A., Ryu C.M., Allen R., Melo I.S. (2007). Rhizobacterial volatile emissions regulate auxin homeostasis and cell expansion in *Arabidopsis*. Planta.

[21] Kundan R., Pant G., Jadon N., Agrawal P.K. (2015). Plant growth promoting rhizobacteria: Mechanism and current prospective. J Fertil Pestic.

[22] Cao H., Jiao Y., Yin N., Li Y., Ling J., Mao Z., Yang Y., Xie B. (2019). Analysis of the activity and biological control efficacy of the *Bacillus subtilis* strain Bs-1 against *Meloidogyne incognita*. Crop Prot..

[23] de Freitas Silva M., Campos V.P., Barros A.F., da Silva J.C.P., Pedroso M.P., de Jesus Silva F., Justino J.C. (2020). Medicinal plant volatiles applied against the root-knot nematode *Meloidogyne incognita*. Crop Prot..

[24] Raza W., Yousaf S., Rajer F.U. (2016). Plant growth promoting activity of volatile organic compounds produced by biocontrol strains. Sci. Lett..

[25] Wheatley R.E. (2002). The consequences of volatile organic compound mediated bacterial and fungal interactions. Antonie Van Leeuwenhoek.

[26] Ryu C.-M., Farag M.A., Hu C.-H., Reddy M.S., Wei H.-X., Paré P.W., Kloepper J.W. (2003). Bacterial volatiles promote growth in *Arabidopsis*. Proc. Natl. Acad. Sci. USA.

[27] Song L., Xu X., Wang F., Wang Y., Xia X., Shi K., Zhou Y., Zhou J., Yu J. (2018). Brassinosteroids act as a positive regulator for resistance against root-knot nematode involving respiratory burst oxidase homolog-dependent activation of MAPKs in tomato. Plant Cell Environ..

[28] Bali S., Kaur P., Sharma A., Ohri P., Bhardwaj R., Alyemeni M.N., Wijaya L., Ahmad P. (2018). Jasmonic acid-induced tolerance to root-knot nematodes in tomato plants through altered photosynthetic and antioxidative defense mechanisms. Protoplasma.

[29] Molinari S., Fanelli E., Leonetti P. (2014). Expression of tomato salicylic acid (SA)-responsive pathogenesis-related genes in Mi-1-mediated and SA-induced resistance to root-knot nematodes. Mol. Plant Pathol..

[30] Nahar K., Kyndt T., De Vleesschauwer D., Höfte M., Gheysen G. (2011). The jasmonate pathway is a key player in systemically induced defense against root knot nematodes in rice. Plant Physiol..

[31] Kyndt T., Nahar K., Haeck A., Verbeek R., Demeestere K., Gheysen G. (2017). Interplay between carotenoids, abscisic acid and jasmonate guides the compatible rice *Meloidogyne graminicola* interaction. Front. Plant Sci..

[32] Kammerhofer N., Radakovic Z., Regis J.M.A., Dobrev P., Vankova R., Grundler F.M.W., Siddique S., Hofmann J., Wieczorek K. (2015). Role of stress-related hormones in plant defence during early infection of the cyst nematode *Heterodera schachtii* in *Arabidopsis*. New Phytol..

[33] Conboy N.J.A., McDaniel T., George D., Ormerod A., Edwards M., Donohoe P., Gatehouse A.M.R., Tosh C.R. (2020). Volatile Organic Compounds as Insect Repellents and Plant Elicitors: An Integrated Pest Management (IPM) Strategy for Glasshouse Whitefly (*Trialeurodes vaporariorum*). J. Chem. Ecol..

[34] Xie S.-S., Wu H.-J., Zang H.-Y., Wu L.-M., Zhu Q.-Q., Gao X.-W. (2014). Plant growth promotion by spermidine-producing *Bacillus subtilis* OKB105. Mol. Plant Microbe Interact..

[35] Pieterse C.M.J., Zamioudis C., Berendsen R.L., Weller D.M., Van Wees S.C.M., Bakker P.A.H.M. (2014). Induced systemic resistance by beneficial microbes. Annu. Rev. Phytopathol..

[36] Farzand A., Moosa A., Zubair M., Khan A.R., Massawe V.C., Tahir H.A.S., Sheikh T.M.M., Ayaz M., Gao X. (2019). Suppression of *Sclerotinia sclerotiorum* by the Induction of Systemic Resistance and Regulation of Antioxidant Pathways in Tomato Using Fengycin Produced by *Bacillus amyloliquefaciens* FZB42. Biomolecules.

[37] Wang J., Zeng G., Huang X., Wang Z., Tan N. (2017). 1,4-Naphthoquinone Triggers Nematode Lethality by Inducing Oxidative Stress and Activating Insulin/IGF Signaling Pathway in *Caenorhabditis elegans*. Molecules.

[38] Kumar S., Kim S., Kim J., Lee J. (2019). Nematicidal activity of 5-iodoindole against root-knot nematodes. Pestic. Biochem. Physiol..

[39] Silva J.C.P., Campos V.P., Barros A.F., Pedroso M.P., Terra W.C., Lopez L.E., De Souza J.T. (2018). Plant Volatiles Reduce the Viability of the Root-Knot Nematode *Meloidogyne incognita* Either Directly or When Retained in Water. Plant Dis..

[40] Gao H., Qi G., Yin R., Zhang H., Li C., Zhao X. (2016). *Bacillus cereus* strain S2 shows high nematicidal activity against *Meloidogyne incognita* by producing sphingosine. Sci. Rep..

[41] Wintermans P.C.A., Bakker P.A.H.M., Pieterse C.M.J. (2016). Natural genetic variation in *Arabidopsis* for responsiveness to plant growth-promoting rhizobacteria. Plant Mol. Biol..

[42] Schulz-Bohm K., Martín-Sánchez L., Garbeva P. (2017). Microbial volatiles: Small molecules with an important role in intra- and inter-kingdom interactions. Front. Microbiol..

[43] Syed-Ab-Rahman S.F., Carvalhais L.C., Chua E.T., Chung F.Y., Moyle P.M., Eltanahy E.G., Schenk P.M. (2019). Soil bacterial diffusible and volatile organic compounds inhibit *Phytophthora capsici* and promote plant growth. Sci. Total Environ..

[44] Anyanful A., Dolan-livengood J.M., Lewis T., Sheth S., Dezalia M.N., Sherman M.A., Kalman L.V., Benian G.M., Kalman D. (2005). Paralysis and killing of *Caenorhabditis elegans* by enteropathogenic *Escherichia coli* requires the bacterial tryptophanase gene. Mol. Microbiol..

[45] Cheng W., Yang J., Nie Q., Huang D., Yu C., Zheng L., Cai M., Thomashow L.S., Weller D.M., Yu Z. (2017). Volatile organic compounds from *Paenibacillus polymyxa* KM2501-1 control *Meloidogyne incognita* by multiple strategies. Sci. Rep..

[46] Schneider J.R., Chadee D.D., Mori A., Romero-Severson D.W.S.J. (2008). NIH Public Access. Bone.

[47] Ryu C.-M.M., Farag M.A., Hu C.-H.H., Reddy M.S., Kloepper J.W., Paré P.W. (2004). Bacterial volatiles induce systemic resistance in *Arabidopsis*. Plant Physiol..

[48] Fincheira P., Parra L., Mutis A., Parada M., Quiroz A. (2017). Volatiles emitted by *Bacillus* sp. BCT9 act as growth modulating agents on *Lactuca sativa* seedlings. Microbiol. Res..

[49] Da Silva J.C.P., Campos V.P., Barros A.F., Pedroso L.A., de Freitas Silva M., de Souza J.T., Pedroso M.P., de Medeiros F.H.V., de Freitas Silva M., de Souza J.T. (2019). Performance of volatiles emitted from different plant species against juveniles and eggs of *Meloidogyne incognita*. Crop Prot..

[50] Oliveira J.T.A., Andrade N.C., Martins-Miranda A.S., Soares A.A., Gondim D.M.F., Araújo-Filho J.H., Freire-Filho F.R., Vasconcelos I.M. (2012). Differential expression of antioxidant enzymes and PR-proteins in compatible and incompatible interactions of cowpea (*Vigna unguiculata*) and the root-knot nematode *Meloidogyne incognita*. Plant Physiol. Biochem..

[51] Zhao P., Li P., Wu S., Zhou M., Zhi R., Gao H. (2019). Volatile organic compounds (VOCs) from *Bacillus subtilis* CF-3 reduce anthracnose and elicit active defense responses in harvested litchi fruits. AMB Express.

[52] Tahir H.A.S., Gu Q., Wu H., Niu Y., Huo R., Gao X. (2017). *Bacillus* volatiles adversely affect the physiology and ultra-structure of *Ralstonia solanacearum* and induce systemic resistance in tobacco against bacterial wilt. Sci. Rep..

[53] Kai M., Piechulla B. (2010). Impact of volatiles of the rhizobacteria *Serratia odorifera* on the moss *Physcomitrella* patens. Plant Signal. Behav..

[54] Bishopp A., Help H., Helariutta Y. (2009). Chapter 1 Cytokinin Signaling during Root Development.

[55] Sang Y., Ryu K.C., Lee S., Bee H., Soo K., Jung H., Lee H., Lee K., Sik W., Hee M.J. (2010). Proteome analysis of *Arabidopsis* seedlings exposed to bacterial volatiles. Planta.

[56] Loon V.A.N., Glick B.R. (2004). 7 Increased Plant Fitness by Rhizobacteria. Mol. Ecotoxicol. Plants.

[57] Sharifi R., Ryu C.M. (2018). Revisiting bacterial volatile-mediated plant growth promotion: Lessons from the past and objectives for the future. Ann. Bot..

[58] Raza W., Shen Q. (2020). Volatile organic compounds mediated plant-microbe interactions in soil. Molecular Aspects of Plant Beneficial Microbes in Agriculture.

[59] Murashige T., Skoog F. (1962). A revised medium for rapid growth and bio assays with tobacco tissue cultures. Physiol. Plant..

[60] Lee B., Farag M.A., Park H.B., Kloepper J.W., Lee S.H., Ryu C.-M. (2012). Induced resistance by a long-chain bacterial volatile: Elicitation of plant systemic defense by a C13 volatile produced by *Paenibacillus polymyxa*. PLoS ONE.

[61] Seo D.-J., Kim K.-Y., Park R.-D., Kim D.-H., Han Y.-S., Kim T.-H., Jung W.-J. (2013). Nematicidal activity of 3, 4-dihydroxybenzoic acid purified from *Terminalia nigrovenulosa* bark against *Meloidogyne incognita*. Microb. Pathog..

[62] Viglierchio D.R., Schmitt R.V. (1983). On the methodology of nematode extraction from field samples: Baermann funnel modifications. J. Nematol..

[63] Gu Y.-Q., Mo M.-H., Zhou J.-P., Zou C.-S., Zhang K.-Q. (2007). Evaluation and identification of potential organic nematicidal volatiles from soil bacteria. Soil Biol. Biochem..

[64] Ntalli N.G., Ferrari F., Menkissoglu-spiroudi U. (2011). Synergistic and antagonistic interactions of terpenes against *Meloidogyne incognita* and the nematicidal activity of essential oils from seven plants indigenous to Greece. Pest Manag. Sci..

[65] Zubair M., Hanif A., Farzand A., Sheikh T.M.M., Khan A.R., Suleman M., Ayaz M., Gao X. (2019). Genetic screening and expression analysis of psychrophilic *Bacillus* spp. Reveal their potential to alleviate cold stress and modulate phytohormones in wheat. Microorganisms.

[66] Jiang C.H., Xie Y.S., Zhu K., Wang N., Li Z.J., Yu G.J., Guo J.H. (2019). Volatile organic compounds emitted by *Bacillus* sp. JC03 promote plant growth through the action of auxin and strigolactone. Plant Growth Regul..

[67] Yang Y.X., Wu C., Ahammed G.J., Wu C., Yang Z., Wan C., Chen J. (2018). Red light-induced systemic resistance against root-knot nematode is mediated by a coordinated regulation of salicylic acid, jasmonic acid and redox signaling in watermelon. Front. Plant Sci..

[68] Velho R.V., Caldas D.G.G., Medina L.F.C., Tsai S.M., Brandelli A. (2011). Real-time PCR investigation on the expression of *sboA* and *ituD* genes in *Bacillus* spp.. Lett. Appl. Microbiol..

[69] Livak K.J., Schmittgen T.D. (2001). Analysis of relative gene expression data using real-time quantitative PCR and the 2-ΔΔCT method. Methods.

[70] Saddique M.A.B., Ali Z., Sher M.A., Farid B., Ikram R.M., Ahmad M.S. (2020). Proline, Total Antioxidant Capacity, and *OsP5CS* Gene Activity in Radical and Plumule of Rice are Efficient Drought Tolerance Indicator Traits. Int. J. Agron..

