# Supplementary material for: Nematicidal Volatiles from Bacillus atrophaeus GBSC56 Promote Growth and Stimulate Induced Systemic Resistance in Tomato against Meloidogyne incognita
Source: Int J Mol Sci. 2021 May 10;22(9):5049. doi: 10.3390/ijms22095049 (PMC8126219; doi:10.3390/ijms22095049)
Supplement: Supplementary file 1 [file ijms-22-05049-s001.zip › ijms-1191284-supplementary.pdf]

Supplementary Material

Nematicidal Volatiles from *Bacillus atrophaeus* GBSC56 Promote Growth and Stimulate Induced Systemic Resistance in Tomato Against *Meloidogyne incognita*

Muhammad Ayaz [1], Qurban Ali [1], Ayaz Farzand [2], Abdur Rashid Khan [1], Hongli Ling [3] and Xuewen Gao [1,*]

[1] Department of Plant Pathology, College of Plant Protection, Nanjing Agricultural University, Key Laboratory of Integrated Management of Crop Diseases and Pests, Ministry of Education, Nanjing 210095, China; m.ayazbiotech@gmail.com (M.A.); qurbanalirattar@webmail.hzau.edu.cn (Q.A.); malix.477@hotmail.com (A.R.K.)

[2] Department of Plant Pathology, University of Agriculture, Faisalabad P.O. Box 38040, Pakistan; ayaz.farzand@uaf.edu.pk

[3] Shandong Vland Biotechnology Co., Ltd, Binzhou, Shandong, 251700, China., linghl@vlandgroup.com

* Correspondence: gaoxw@njau.edu.cn; Tel: +86-025-8439-5268

Table S1. Primers used in the study.

Gene description	Code	Primers	Sequence
1-aminocyclopropene-1-Carboxylate Oxidase	*ACO1*	*ACO1*-F<br>*ACO1*-R	GATCAAGGGACTCCGCGCTC<br>TGGCGCATGGGAGGAACATC
cytokinin oxidase/dehydrogenase-like	*SLCKX1*	*SlCKX1*-F<br>*SlCKXI*-R	AACCTAGGTGGGTGGGGTGG<br>CAACTTCCGCCGCTTTTCCG
Auxin synthesis gene	*SlIAA1*	*SlIAA1*-F<br>*SlIAA1*-R	AAACCCCACCACCTGTTGCC<br>GCAGGGGCAAATTCAGAGCC
Expansin synthesis gene	*exp18*	*exp18*-F<br>*exp18*-R	GGTGGAGCGTGTGGGTATGG<br>GGAGGGTTACACCAACCGCC
Pathogenesis related protein 1	*PR1*	*PR1*-F<br>*PR1*-R	GGTGGCCGACCACAACCTAC<br>GCCGTGCAATTGTGGGTGTC
Pathogenesis related protein 5	*PR5*	*PR5*-F<br>*PR5*-R	TGGTGGTTTTGGGCTCCACC<br>CATTTTCCCGGCCCAGGGTT
Lipoxygenase 1	*SlLOX1*	*SlLOX1*-F<br>*SlLOX1*-R	CCGCGAACCAACCAAAGCAG<br>ACCAAGGGCCTTCAGGCAAC
